# The pursuit of accurate predictive models of the bioactivity of small molecules

**DOI:** 10.1039/d3sc05534e

**Published:** 2024-01-12

**Authors:** Karina Martinez-Mayorga, José G. Rosas-Jiménez, Karla Gonzalez-Ponce, Edgar López-López, Antonio Neme, José L. Medina-Franco

**Affiliations:** a Institute of Chemistry, Merida Unit, National Autonomous University of Mexico Merida-Tetiz Highway, Km. 4.5 Ucu Yucatan Mexico kmtzm@unam.mx; b Institute for Applied Mathematics and Systems, Merida Research Unit, National Autonomous University of Mexico Sierra Papacal Merida Yucatan Mexico; c Department of Theoretical Biophysics, IMPRS on Cellular Biophysics Max-von-Laue Strasse 3 Frankfurt am Main 60438 Germany; d Department of Chemistry and Graduate Program in Pharmacology, Center for Research and Advanced Studies of the National Polytechnic Institute Mexico City 07000 Mexico; e DIFACQUIM Research Group, Department of Pharmacy, School of Chemistry National Autonomous University of Mexico Mexico City 04510 Mexico

## Abstract

Property prediction is a key interest in chemistry. For several decades there has been a continued and incremental development of mathematical models to predict properties. As more data is generated and accumulated, there seems to be more areas of opportunity to develop models with increased accuracy. The same is true if one considers the large developments in machine and deep learning models. However, along with the same areas of opportunity and development, issues and challenges remain and, with more data, new challenges emerge such as the quality and quantity and reliability of the data, and model reproducibility. Herein, we discuss the status of the accuracy of predictive models and present the authors' perspective of the direction of the field, emphasizing on good practices. We focus on predictive models of bioactive properties of small molecules relevant for drug discovery, agrochemical, food chemistry, natural product research, and related fields.

## Abstract 2

Each step in model development affects its accuracy.

## Graphical abstract

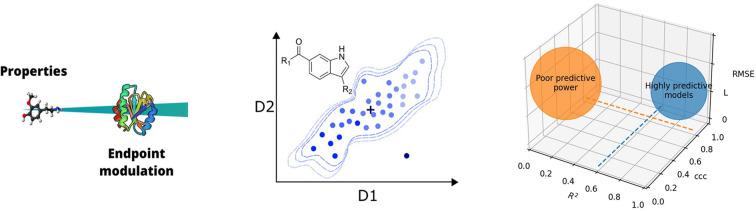

## 1 Introduction

Forecasting and predicting events reside in human nature. From a philosophical point of view, the capability to make useful and accurate predictions gives humans a sense of control. Moreover, humans are aware of rare events (aka, “black swans”) which are difficult or nearly impossible to predict. Yet, rare events play crucial roles in different fields.^1^ As discussed later in this perspective, in science, rare events have received different names, such as anomalies, outliers, atypical values, or property cliffs.

Gathering experimental information is essential but can be very costly, time-consuming, environmentally or animal-unfriendly, or even impossible to perform. Furthermore, some experiments might be risky, pose safety issues or be unethical, *e.g.*, doing experiments on animals or in humans.^2^ In turn, computational models and mathematical predictions have practical importance, they can substitute unfeasible or inconvenient physical experiments or prevent life-threatening events. Thus, it is important to have predictive models in place to quickly respond to emergencies. In public health, the recent COVID-19 pandemic, clearly showed the need for swift development of vaccines, drugs, and detection methods. Notably, statistical, and predictive models were key for the successful delivery of vaccines to contain the effects of the virus.

**Fig. 1:**
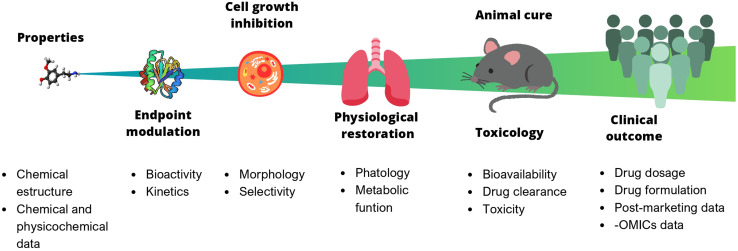
Predictive models of bioactivity depend on the size and complexity of the system under study. Each stage can be described by different properties. Properties of larger and more complex systems (far right) are harder to get, more costly and more difficult to predict.

In chemistry, anticipating properties is a common practice, *e.g.*, predicting reactivity, spectroscopic information, synthetic feasibility, stability in materials, toxicity, and biological activity, to name a few. In multidisciplinary areas such as drug discovery, it is of utmost interest to predict biological activity, toxicity (at different levels, from cells to animals to humans), bioavailability, and pharmacokinetic properties. Predictive models of bioactivity strongly depend on the size and complexity of the system under study, as depicted in Fig. 1. The systems range from a relatively simple assays *e.g.*, binding affinity, to larger and more complex ones, such as cell-based assays, animal models, or even clinical trials. Early stages in drug discovery campaigns start with small, fast, simple, and relatively cheap experiments. As the biological system increases in size, the time of exposure and the number of non-controllable factors also increases. For example, the number of variables involved, the variability, and the errors. As a result, it becomes nearly impossible to consider all the underlying variables influencing the property to be predicted. Undeniably, data reductionism is needed, but it is important to keep in mind that this affects the scope of the study and might impact inferences made based on those models.^3^

Predictive models are meant to be reusable. However, updates are needed when new data opposes the original predictions or to extend the applicability domain originally covered. Key components in the development of predictive models include the need to validate its predictive power; set criteria to assess the quality of a predictive model; evaluate the confidence of the predictions; or even decide if the model is reliable enough to make decisions.

Chemical informatics, chemoinformatics or, also referred in the literature as cheminformatics,^4^ relies on the Data Information Knowledge Wisdom Pyramid (DIKW) paradigm.^5^ Under such paradigm, predictive models can be seen as generalizations and contribute directly to knowledge. Predictive models should be subject to refinement, as part of an iterating process of generation, application, and refinement. In the current era of big data, where data is increasing daily and at an unprecedented rate^6,7^ comes the need to constantly generate new models and refine or update existing ones. Exploration of larger amount of data not only makes the prediction of properties possible but also has make apparent huge data gaps that need attention and the suitability of further development of predictive models.

Along with the development of predictive models (a form of artificial intelligence), approaches and metrics to statistically assess the performance and practical value of the models has evolved. Ultimately, those metrics allow the comparison of the outcomes, regardless the methods used. Initially, the focus was in the internal validation methods. As the data was further analyzed and confronted with compounds not used in the development of the models (external validation), it became obvious that internal validation was not enough, and external validation was then deemed necessary.^8^ As will be described later in this perspective, we are now at the point where other metrics, such as the mean absolute error (MAE) and the root mean squared error (RMSE), are recommended for complementing the assessment of the accuracy of predictive models. Interestingly, researchers from other fields have landed on the same ground. For example, in Environmental Sciences, Li^9^ proposed the metric “variance explained” (VE) to measure the accuracy of predictive models.

As discussed in the literature, arguably one of the most simplistic predictive models of chemical properties relies on the similarity principle, *e.g.*, compounds with similar structures have similar properties. Such a basic and intuitive principle has at least two significant challenges, namely, how to unequivocally measure similarity, and how to deal with similar chemical structures that have large and unexpected property differences *e.g.*, “property cliffs”^10^ and activity cliffs.^11–13^ The former point is intimately related to molecular representation that defies any computational applications, and it is at the core of chemoinformatics. The second hurdle, property cliffs, brake down predictive models. Interestingly, property cliffs receive different names in different areas such as rare events and anomalies, or “black swans”. As commented hereunder, rare events could be one of the most useful or more significant events (results).

The goal of this manuscript is to provide a critical assessment of the status of the accuracy of predictive models and present the author's perspective of the direction of the field, commenting on good practices. The perspective focuses on predictive models of bioactivity of small molecules relevant for drug discovery, and agrochemical- and food-chemistry. In addition, we will highlight unresolved issues that merit attention, such as statistical parameters to assess predictivity and malpractices that can and should be addressed.

## 2 Present

Each step involved in the development of predictive models impacts the accuracy of the prediction. For example, the quality and quantity of the input dataset, the selection of significant descriptors, the appropriate splitting of the data, the statistical tools used, *etc.*^14^ This section summarizes important aspects to consider before, during, and after to develop predictive models and how this influences accuracy. The discuss includes data preparation and selection, experimental design, applicability domain, and the assessment of accuracy.

### 2.1 Data preparation and selection

#### 2.1.1 Number and diversity of molecules in databases

A compound dataset is required to develop a model. Ideally, such a dataset should contain structurally diverse molecules and should cover a wide range of values of the target property. Structurally diverse datasets capture a broad range of structural features that ultimately will provide a larger applicability domain. Those models will learn better; generalize the underlying structure–property relationships; and decrease bias towards specific chemical classes or structural motifs.

Historically, the first Quantitative Structure–Property Relationships (QSPR) models were developed with a series of analogs with slight chemical variations (local models). Local models are usually generated from small datasets, containing from tens to hundreds of molecules. A key concept to analyze structural diversity, among other properties, is the chemical space, which can be defined as the *n*-dimensional space that defines the molecules under study. Local models occupy constrained regions of chemical space, were, typically, small variations in structure lead to small variations in activity or property. Frequently, those datasets can fit linear models. However, even simple models are not exempt of having activity cliffs. A property (activity) cliff is a pair of compounds with high structure similarity (*e.g.* based on their structural or physicochemical profiling and a similarity metric) but large property (biological activity) difference.^15^ A traditional form to identify property and activity cliffs is the Structure–Activity Landscape Index or SALI value. Which is a ratio of the activity difference of a compound's pair over the distance or inverse similarity.^16^ For example, compounds with SALI values higher than two standard deviations (concerning the data set's average SALI value) are considered an activity cliffs.^17^ Also, novel classification methodologies based on QSAR models allow the systematic identification of activity cliffs from large datasets.^18^ Additional quantitative and graphical methods to identify activity cliffs include structure-activity similarity maps, structure–activity relationship index-SARI, network-like similarity graphs, dual activity difference maps, combinatorial analog graphs, and similarity-potency trees which has been reviewed elsewhere.^19^ Therefore, analysis of activity cliffs should be performed on each QSPR model.

Global models are made with medium and large datasets with hundreds or thousands of molecules. Molecular diversity is key to generating global models. Also, by including diverse compounds, the model can mitigate data gaps and biases that may arise due to the underrepresentation of particular chemical classes or activity ranges. This promotes a more balanced and comprehensive understanding of the relationship between chemical structure and property.

Molecular databases with low diversity usually contain congeneric compounds with small structural variations at specific points. These smooth changes in structure may lead to small variations in activity or property, making them suitable to model with “simple” linear models. As expected, problems may arise if activity cliffs are present in the database.

#### 2.1.2 Data imbalance, inactive compounds and the need for negative data

A survey on ChEMBL V.29, shows that only 11% of the registered biological targets have a balanced dataset (same proportion of active and inactive compounds), and all the others have a higher proportion of active (58%), or a higher proportion of inactive molecules (31%). This survey highlights the need of publishing “active” and “inactive (negative)” data. The bias towards reporting active molecules might be the result of projects oriented to obtaining lead molecules, which is a valid goal if, for instance, those molecules are moved forward in drug discovery pipelines. However, for enriching the chemical space explored for a particular endpoint, for the development of predictive models, or for describing structure–activity relationships, the inclusion of inactive molecules is needed. Data imbalance could be gradually overcome, as we reassess the inactive data. Inactive or negative data is valuable and should not be perceived as useless. As a scientific community we are called to use, analyze, and publish active as well as inactive data. Initiatives that pay special care to the design of the databases, such as Tox21 (ref. 20) are a reference of good practices.

Also, there are cases with a minimum quantity of data *e.g.*, emerging, rare, or neglected diseases that must be complemented with data augmentation algorithms or decoys.^21,22^ In these instances, the validation of the data augmentation algorithm or the decoy generation method is essential. For example, it is important to analyze structural and chemical diversity, chemical space coverage, drug- or lead-likeness, and similarity to active compounds or positive controls. These practices could avoid generating unrepresentative negative/inactive chemical structures that impact the accuracy and usefulness of predictive models.

#### 2.1.3 Experimental variability

Data quality is key in the development of predictive models. Data quality intrinsically refers to the precision and reproducibility of the data obtained. However, in real practice, multiple environmental, social, or biological conditions change constantly,^23,24^ which generates variability in the experimental measurements even if the same research group uses the same protocol or if separate groups employ similar (but no identical) protocols.^25^ Despite these known sources of variability, it is commonly inadvertently ignored. Model developers need to assess the data quality, and data generators should provide all the required information to make such assessments.

Experimental data should be obtained using standardized protocols and quality controls to minimize errors and inconsistencies. Standardization of data is necessary to ensure compatibility and comparability. This includes standardization of molecular representations, activity values, and descriptor calculations. It is important to ensure the reliability of experimental measurements, such as activity or property values, to avoid introducing, or at least reduce as much as possible, noise or bias into the models.

The biological information is derived from a variety of experiments involving different systems such as proteins, enzymes, cells, organisms or animals. In addition, the measurements may be focused on bioactivity, binding affinities, toxicity, among others.

As instrumentation and methodologies evolve it influences the precision, sensitivity, and specificity of measurements, thereby contributing to the observed numerical differences in the data. Thus, the selection of a particular technology can significantly influence data quality and introduces another layer of variability. Notably, the experimental biases, errors arouse from different laboratories and instrumentation used, needs to be considered as a pretreatment of the data.

Predictive models of bioactivity are interdisciplinary by nature, it often involves collaboration among research laboratories with distinct methodologies, equipment, and personnel. The lack of standardized procedures introduce variability in experimental conditions, affecting reproducibility of the results. Standard Operating Procedures (SOPs) establishes guidelines that researchers across different laboratories can adhere to, thereby ensuring consistency in experimental workflows.^26^ Moreover, SOPs contribute to the harmonization of data collection, preprocessing, and analysis, fostering a shared understanding of best practices within the cheminformatics community. This not only streamlines collaborative efforts but also enhances the collective ability to validate and build upon each other's work.^27^ One of the primary advantages of incorporating SOPs into cheminformatics research is the heightened transparency they bring to experimental conditions. The explicit documentation of procedures, reagents, and instrumentation fosters a clear and detailed account of each step in the experimental process. This transparency not only aids in the understanding of methodologies by peers but also facilitates the reproduction of experiments, a cornerstone in science. The predictive power of numerical models largely depends on the consistency of the input data. SOPs can provide a systematic approach to data generation and analysis, reducing the likelihood of unintended variations that may compromise the integrity of the models. Thus, adopting SOPs is imperative to ensure consistency, transparency, and reproducibility.^28^ This commitment to standardized procedures establishes the basis for robust, reliable, and impactful cheminformatics research at large.

#### 2.1.4 Stereochemistry and tridimensionality: a chemical challenge and a structural beauty

Stereochemistry is by itself one of the most challenging aspects of molecular design. The specific configuration around stereocenters largely impacts biological and toxicological profiles. Therefore, QSP(A)R models should predict activity differences between stereoisomers. To generate stereochemistry-sensitive predictive models, there are two important requirements that must be fulfilled, namely proper molecular representation and data availability. Suitable molecular representations would detect the configuration of stereocenters. The model developer must be aware that many descriptors traditionally used in two-dimensional Quantitative Structure–Activity Relationships (2D-QSAR) models, based on connectivity, atomic properties, and graph theory, are usually blind to stereochemical information. There are, however, sets of descriptors and other representations that can distinguish enantiomers: based on classical connectivity and topological analysis, for example the chirality-corrected descriptors proposed by Golbraikh, Bonchev and Tropsha,^29^ the physicochemical atomic stereodescriptors (PAS),^30^ or the simplex representation of molecular structure (SiRMS).^31^ Novel approaches using machine learning such as Graph Neural Networks (GNN) have also being explored.^32–34^ The developer should assess whether those descriptors are available in the selected software for modeling. Alternative approaches that can naturally be sensitive to stereochemistry are CoMFA or CoMSIA methods. These methods depend on the conformation of molecules and, since the biologically active conformers are usually unknown, combination with other approaches like docking or 3D alignment to experimental structures is strongly recommended.

The second requirement for a stereo-sensitive predictive models is, as in any data-driven method, the availability of stereochemical information in the data sources. Commonly, the generation of the datasets for modeling is done by retrieving the information deposited in public databases such as ChEMBL,^35^ PDBbind,^36^ or PubChem BioAssays.^37,38^ According to the ChEMBL database, as reported by the chirality filter implemented in the website,^39^ at the time of this publication (release 33), there are 2 399 743 registered compounds. From this, only 6035 (0.25%) are achiral molecules, and from the set of structures with chiral centers, 8034 (0.33%) are specific enantiomers, 2685 (0.11%) are racemic mixtures, and 2 382 989 (99.31%) have unknown chirality.

After consulting the original sources cited in the database, there are several reasons why stereoisomers are not specified. In early stages of the discovery of bioactive molecules, high throughput experiments are performed to explore a wide region of the chemical space with the purpose of identifying novel chemotypes with a particular activity. Therefore, the specific enantiomer is usually not a priority at this step. In contrast, during structure–activity relationships exploration of hits and hit-to-lead optimization campaigns, both the objectives and the experimental design are substantially different from high throughput experiments. Some publications about optimization and SAR exploration report activity for a single enantiomer or for racemic mixtures, while other enantiomers were not explored, or their activity could not be measured with the same experimental protocol. It is also common to find discrepancies in activity when molecules identified in high throughput campaigns are re-evaluated in a low throughput biological assay. As a consequence, data generated from these different sources is hardly comparable. Table 1 shows examples of molecules found in ChEMBL and PubChem BioAssays. Those molecules have stereocenters and were evaluated on different biological assays, however, enantiomeric information is not included.

**Table 1:** Examples of molecules without specified stereochemistry in ChEMBL and PubChem BioAssay databases

Structure	ChEMBL/PubChem BioAssay ID	Activity/target	Comment	Reference
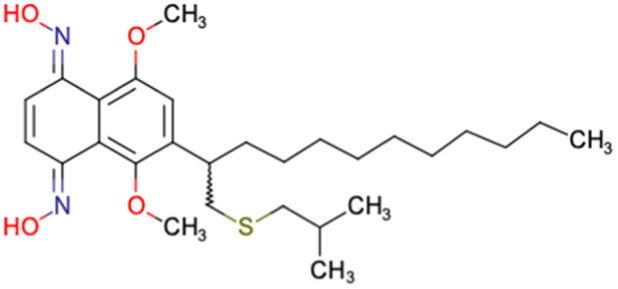	CHEMBL4589953	Cytotoxicity against HCT-15, MGC-803, K562, and HSF cell lines	No discussion about stereochemistry	32
			SAR exploration	
			Low throughput biological data	
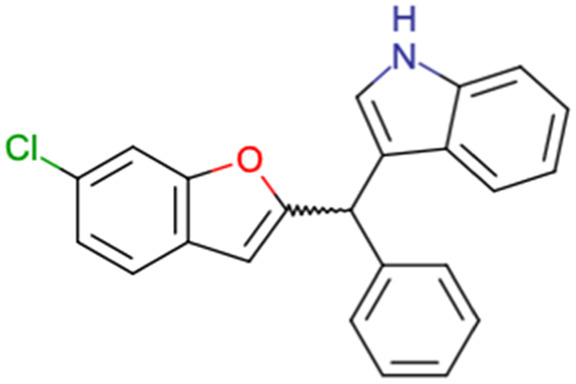	CHEMBL4753528	Cytotoxicity in SiHa and C33a cells	No stereochemistry is discussed	33
			SAR exploration	
			Low throughput biological data	
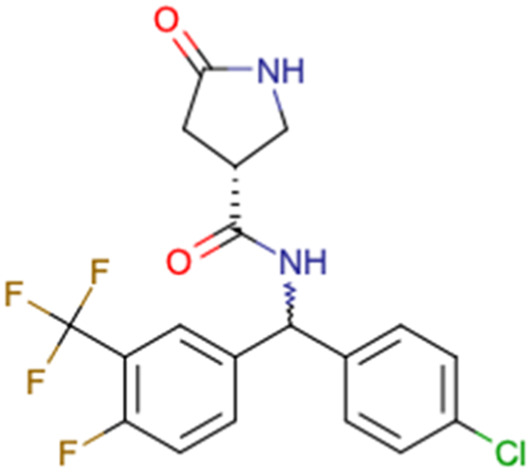	CHEMBL5196342	Inhibitory activity against Na_*v*_1.8 voltage-gated sodium ion channels	Racemic mixtures are obtained after synthesis	34
			Low throughput biological data	
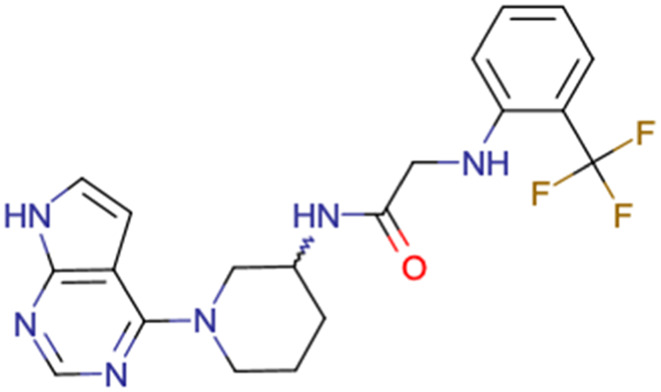	CHEMBL5188351	Inhibitory activity against Bruton's tyrosine kinase	Hit-to-lead optimization process	35
			No stereochemistry discussed in the first stages of experiments	
			Stereochemistry is included in the late optimization process	
			Low throughput biological data after virtual screening	
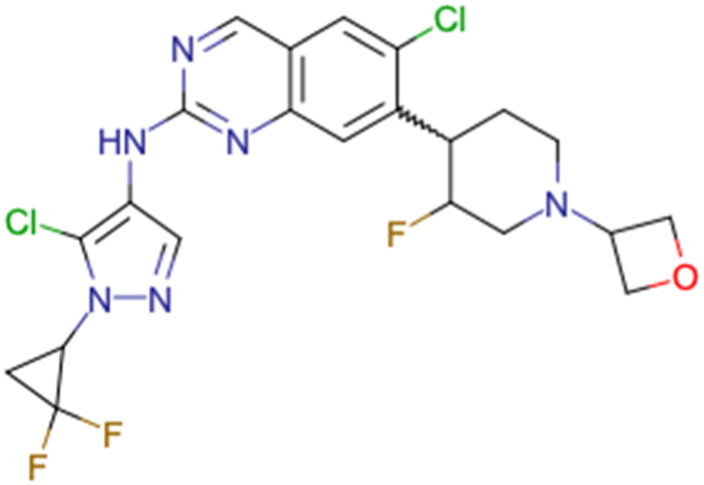	CHEMBL5070231	Inhibitory activity against the leucine-rich repeat kinase 2 (LRRK2) and some other pharmacokinetic activities	Hit-to-lead optimization process	36
			No stereochemistry discussed in the first stages of experiments	
			Stereochemistry is included in the late optimization process	
			Low throughput biological data after virtual screening	
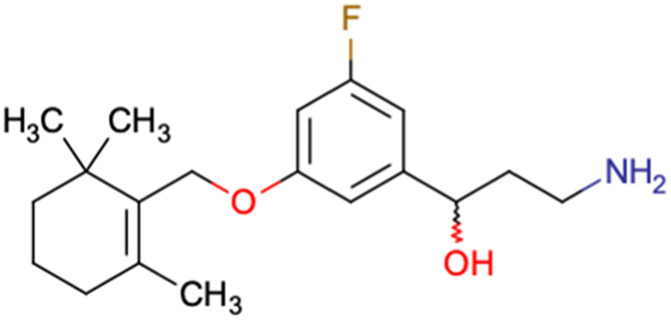	CHEMBL5086566	Inhibitory activity against retinoid isomerase (RPE65)	Pure enantiomers and racemic mixtures are tested	37
			SAR exploration	
			Low throughput biological data	
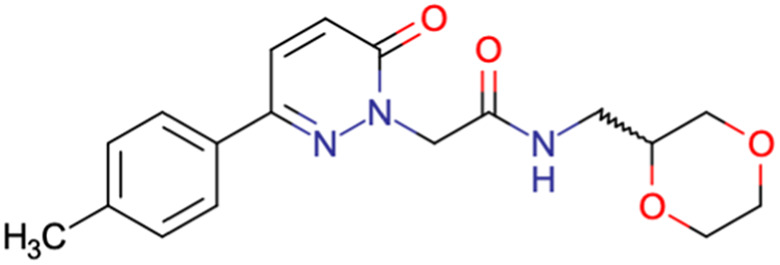	SID49725781	Inhibition of apicoplast development in *Plasmodium falciparum*	High-throughput screening data (1280 compounds)	38
			The main goal of this assay is finding molecules with potential anti-malarial activity	
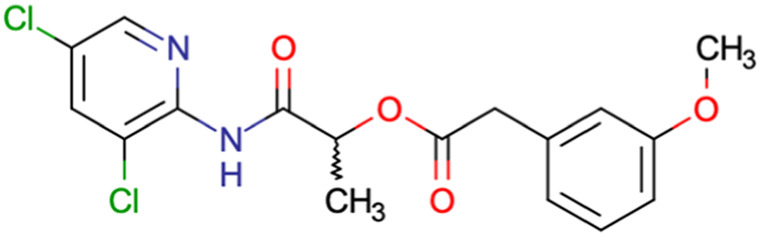	SID56324664	Inhibition of active B-cell receptor	High-throughput screening data (1280 compounds)	39
			The main goal of this assay is finding molecules with potential activity against B-cell lymphoma	
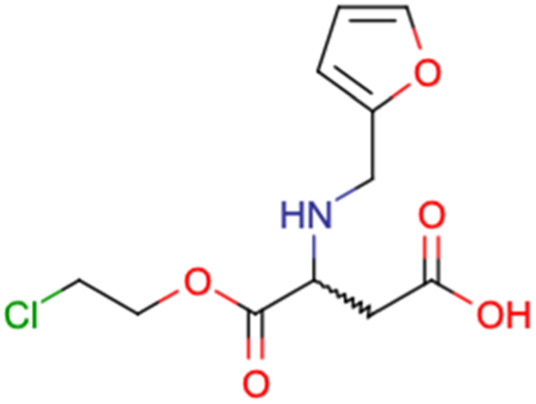	SID24821117	Inhibitory activity against polymerase iota	High-throughput screening data (391 277 compounds)	40
			The main goal of this assay is finding potential inhibitors of polymerase iota	
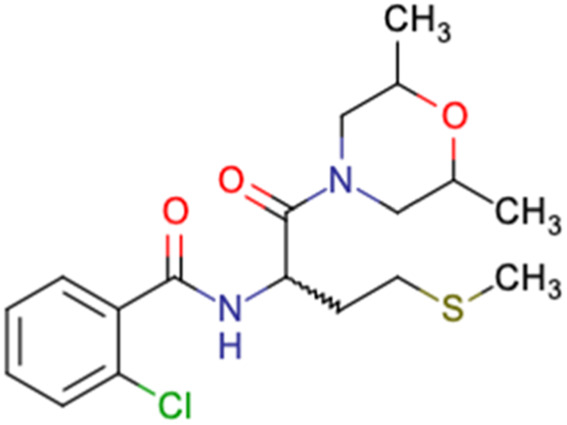	SID57257818	Nuclear DNA content in adenocarcinoma cells	High-throughput screening data (344 074 compounds)	41
			The main goal of this assay is finding chemical families with potential antiproliferative activity on adenocarcinoma cells	

Clearly, the inclusion or omission of stereochemistry in a predictive models of bioactivity is not a trivial decision.^50^ A critical assessment of stereochemical information should be part of the data curation process and must be discussed in any report. As shown before, a diagnosis of the available data helps to make critical decisions for future steps, for example, in the choice of descriptors or the machine learning algorithm for modeling.

### 2.2 Experimental design

#### 2.2.1 Local *vs.* global models

**Fig. 2:**
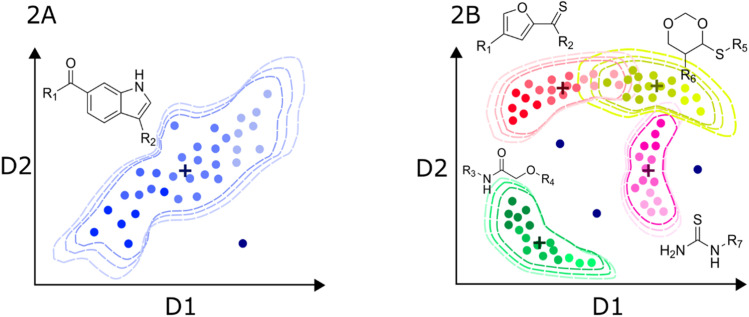
Hypothetical distribution of a local (A) and global (B) predictive models of bioactivity. Axis labels D1 and D2 represent the potentially multidimensional set of descriptors relevant for activity. Dashed contours represent the limits of the applicability domain in chemical space. Color intensity denotes the activity trends in the datasets. Putative data centroids are marked with a cross. Outliers are also depicted as dark blue dots outside the applicability domain limit.

As discussed above, chemical diversity and balance in the modeling dataset is the main factor that defines the extent of the chemical space covered by the predictive models, and its scope, local or global. Fig. 2A shows a schematic representation of a hypothetical low-diversity, local dataset. In this type of data, molecules are commonly distributed in a well-defined region of the chemical space, where the borders are quantitatively set using the applicability domain method chosen by the modeler. Since the information contained in local datasets tends to be more homogeneous, structure–activity/property trends are often smooth. The concept of “applicability domain” has several definitions and each definition serve different purposes. For example, to assess the validity, confidence, and decidability. Moreover, the method employed to assess the applicability domain depends on the type of information available. Excellent reviews discussing this topic are available in the literature.^51,52^

Since the information contained in local datasets tends to be more homogeneous and structure–activity/property trends are often smooth. Models generated with this type of data are usually highly predictive. Also, because linear models are easier to interpret, local models may provide valuable insights into the underlying biological or physico-chemical mechanisms that govern the behavior of the target variable. One of the main disadvantages is the poor generalization to other chemical families.

In turn, the presence of different chemotypes with very different atom connectivity and variety in sidechains, are structurally diverse and potentially leads to more complex activity/property trends. Therefore, the use of nonlinear algorithms is often required to appropriately model the behavior of the independent variable as a function of the structure. Fig. 2B illustrates a representation of a high-diversity dataset used to build a global model. In this case, structural changes are more abrupt, and they are not limited to chemical substitutions at specific points but even the chemical scaffold is allowed to change. Consequently, the distribution of molecules in the chemical space is more complicated, and the boundaries are also difficult to define. Ideally, the machine learning algorithms should identify the chemotype of each molecule and apply the trends learned from the data. In practice, molecules from different chemotypes could overlap in the space of descriptors relevant to the activity/property. This overlap may originate “confusion” in the model, affecting its predictability. Furthermore, the inhomogeneous distribution of molecules could originate from regions of high and low density or even “holes” of information inside the general space covered by the model.^53^ Therefore, it is important that the different chemical families in the database have appropriate populations to ensure an equilibrated representation of diverse molecules. Thus, singletons should be avoided, *i.e.*, unique molecules with large structural differences to any other compound in the set. In this regard, clustering and diversity analysis are encouraged prior to modeling.^54^

Another important issue with global models is the definition of the applicability domain. As schematically depicted in Fig. 2A and B, there are remarkable differences in the distribution of molecules in the chemical space. One critical difference in diverse data sets is the formation of clusters around specific scaffolds or chemotypes with different local activity trends. In Fig. 2A and B, centroid positions are marked with a cross. For a local model, such as the hypothetical dataset in Fig. 2A, distance-from-centroid methods are useful for the applicability domain definition because the training molecules tend to form a single cluster in the space of chemical descriptors. In contrast, molecules in the training sets of global models often form several, potentially overlapping clusters. The distances of the molecules to the centroid of the whole dataset cannot distinguish the presence of holes or low-density regions, where predictions are not expected to be accurate. For this reason, density or clustering-based methods are preferred to set the limits of the applicability domain of global models. Excellent reviews and detailed descriptions of different methods for applicability domain definition can be found in the literature.^55–57^

#### 2.2.2 Screening *vs.* design

Predictive models are mainly used for virtual screening or design purposes. Since virtual screening is an early step in molecular design where molecular diversity plays an important role. Thus, for virtual screening, global models are more appropriate (*vide supra*). In turn, local models are more suitable for capturing smaller structural changes required for molecular design (*e.g.*, hit-to-lead optimization). Even though accuracy is decided in both circumstances, for screening purposes, one can be less rigorous at the screening stage, in terms of accuracy. When looking for new and diverse molecules, it is better to try many rather than fewer molecules. Complementary, if design is pursued, one should aim for accurate predictions with less chance of error.

#### 2.2.3 Consensus approaches

Consensus strategies are based on the premise that the fusion of several models reduce the prediction uncertainty, increase the classification performance, and overcome limitations of individual predictive models.^58,59^ This strategy offers the opportunity to generate, analyze, and sample data with different perspectives. In consensus QSAR methods, multiple data observations or calculations (descriptors) are combined to increase the accuracy of the predictions.^60^ Different consensus models can be developed depending on the type of information analyzed:

(1) Multiple independent models of the same dataset. Predictions based on the consensus of several QSAR models renders more accuracy, compared to the selection of single “best” model. Additional information further increase accuracy, such as the incorporation of read-across outcomes. For example, a consensus model to analyze soil ecotoxicity data involving several QSAR models and read-across toxicological data^61^ give better accuracy than individual models. Similar comparisons, but for classification tasks of large-scale datasets has been analyzed,^62^ and the incorporation of chemical space analysis has been also reported.^63^

(2) Combination of QSAR models of different endpoints: each model contributes with features (information) that is relevant for the prediction of activity of new molecules. This area is also known as multitasking learning. For example, for aquatic toxicity data, multitask random forest outperformed individual models. In this study, knowledge shearing between species was key for the development of multitask models.^64^

Consensus approaches create a broader view of a complex system, improving the accuracy of conventional predictive models.^65,66^

### 2.3 Representation, feature selection, and applicability domain

To pursue accurate and reproducible predictive models, the independent variables should be carefully obtained and selected. In chemistry-related models, independent variables are usually molecular descriptors that can be physically measured or, most commonly, calculated. There is a variety of molecular descriptors, and they can be calculated with different software programs. The descriptors employed define the chemical space. The descriptor-dependency of the chemical space concept characterizes and distinguishes the cheminformatics field.^67^ Thus, a set of molecules can be defined by different sets of descriptors that collectively can be referred to as “chemical multiverse”.^68^

Frequently, for the generation of predictive models, the descriptors are chosen based on feature selection methods such as genetic algorithms,^8^ particle swarm optimization,^69^ principal component analysis,^70^ among many others. To note, deep learning methods do not necessarily require a feature selection step.

The smaller number of descriptors used for the predictive model, the better. Each selected descriptor should capture the variability of the targeted property in a unique and not redundant manner. Thus, a poor selection of descriptors leads to models with low predictivity or the lack thereof. Once the descriptors are selected, a further check commonly employed is the chance correlation through Y-scramble or Y-randomization methods.^71^

Importantly, to care about reproducibility and further use of the models, full documentation of the descriptor calculation and selection should be provided (Table 2).

**Table 2:** Models developed with main/classical molecular descriptors: standard and non-standard

Endpoint modeled	Standard descriptors	Non-standard descriptors
Structure–property relationships	• Classical molecular fingerprints (*e.g.*, MACC keys, PubChem, ECFP)	• Non-classical (and recently developed) molecular fingerprints (*e.g.*, MAP4, and atom pairs)
	• Chemical diversity descriptors (*e.g.*, functional groups and Bemis–Murko scaffolds)	
Industrial applicability	• Druglike properties (*e.g.*, log *P*, molecular weight)	• Organoleptic properties (*e.g.*, odor or flavor)
		• Material properties (*e.g.*, conductivity)
ADMET predictions	• Qualitative ADMET descriptors (*e.g.*, inhibitor of cytochromes)	• Quantitative ADMET descriptors (*e.g.*, clearance, bioavailability, half-life time)
Reactivity	• Polarizability	• Quantum descriptors
Biological and bioactive	• Bioactivity (*e.g.*, enzymatic or cell grown inhibition)	• Post-marketing data (*e.g.*, drug safety in different populations)
	• Phenotypic effects	• Omic data (*e.g.*, pharmacogenomic or proteomic data)

Lastly, the set of descriptors employed in the models defines its applicability domain. Since mathematically the applicability domain can be defined and calculated in different ways, it is a good practice to analyze the applicability domain with different methods.

### 2.4 Assessing accuracy

After the generation and selection of models that can describe the structure–activity/property trends in the training data, one of the most critical aspects to address is to prove the generalization of the model to data never used for its generation, within the limits of the applicability domain. Interestingly, this question has been at the center of discussion in the QSAR/QSPR community since the early days of its history. Nowadays, there is still debate about how to test if a model is predictive and if the error calculated from known data is also expected in the real use of the model, when new molecules are synthesized or tested for the desired activity. After all, as with any scientific theory, the trust in a model relies on its ability to predict new data. In this regard, the proper use of statistical methods is an essential requirement when reporting or publishing predictive models. The standard practice for evaluating model predictivity is to split the original data prior to modeling, reserving at least one of the partitions to predict the activity or property of the molecules in this set with the final model. Thus, the extent of calculated error of the molecules in the test set is assumed to be the expected uncertainty of the predictions for new molecules. The most commonly used metrics to assess model performance are based on the coefficient of determination, *R*^2^, and on the average error made by the model, such as RMSE, defined in equations shown in Table 3. Metrics based on *R*^2^ are commonly referred to as the *Q*_F*n*_^2^ family of parameters. Unfortunately, lack of consistency on the nomenclature of the validation parameters is still a problem and the use of the common symbol *R*^2^ is a frequent practice. QSAR developers should strictly adhere to a unified nomenclature scheme to facilitate communication and avoid misinterpretations.

**Table 3:** Recommended external validation parameters to test the predictivity of QSAR/QSPR models. All parameters listed are calculated using the data from the external test set^a^

Validation statistics	Formula	Interpretation
Coefficient of determination	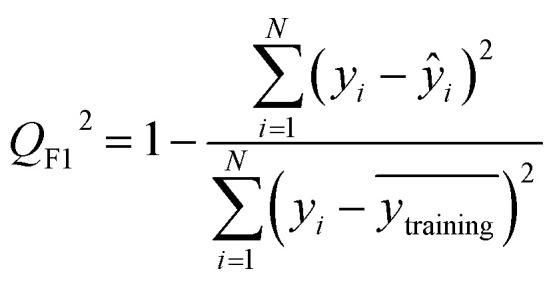	Proportion of the variance that can be explained by the model
	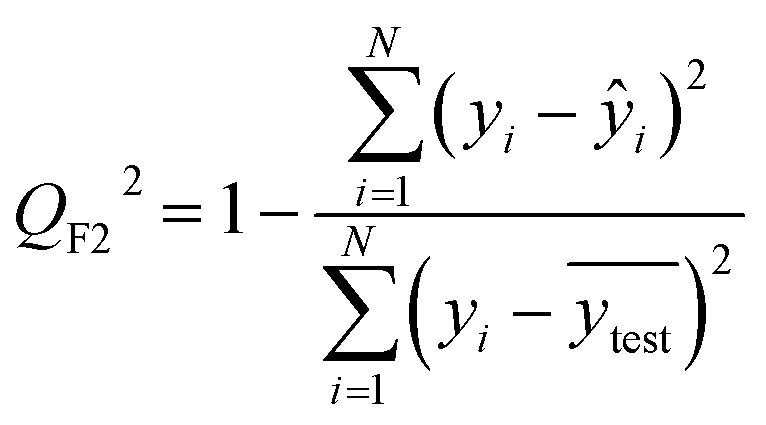	
Root mean squared error	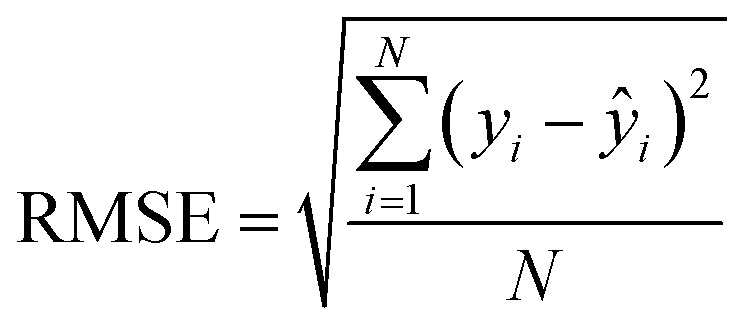	Observed average error made by the model
Mean absolute error	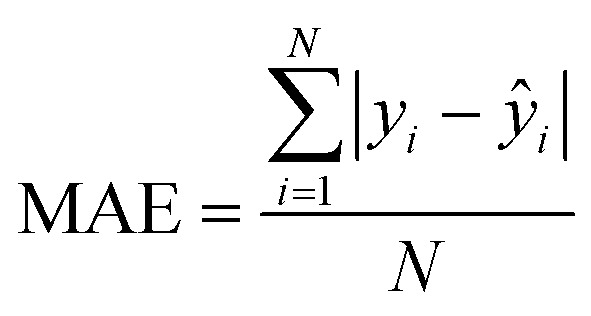	Observed absolute average error made by the model
Concordance correlation coefficient	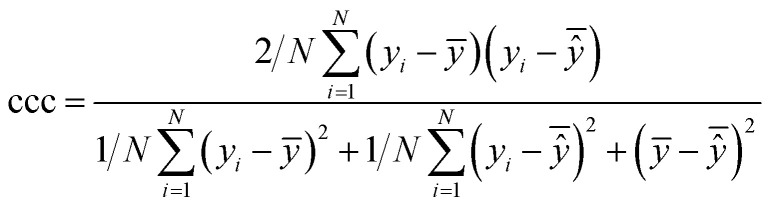	Extent of agreement between two random variables (in this case, the experimental and predicted values)
Accuracy	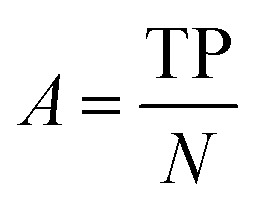	Fraction of correct predictions
Recall	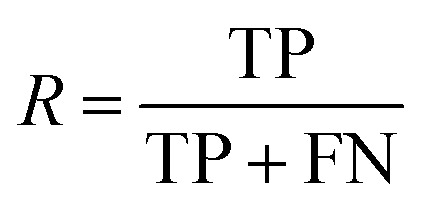	Fraction of molecules in a class that could be correctly predicted
Precision	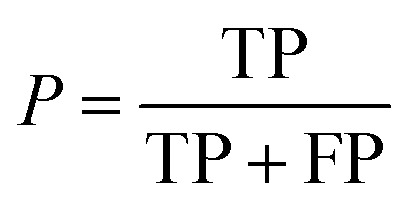	Fraction of correct predictions in a class
Area under the receiver operating characteristic curve (ROC)	Calculated by integration of the ROC curve	The ROC curve is the plot of the false positive rate *vs.* the true positive rate. A perfect classifier has a total area of 1
Matthews correlation coefficient	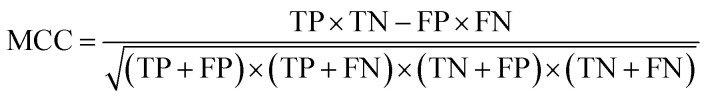	Correlation between the true and predicted classes

a *y*_*i*_, experimental activity/property of molecule *i*; *ŷ*_*i*_, predicted activity/property of molecule *i*; *ȳ*, average of experimental activities/properties; 
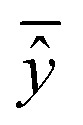
, average of predicted activities/properties; *N*, number of molecules, TP, true positives; FP, false positives; TN, true negatives; FN, false negatives.

The *Q*_F*n*_^2^ family is often preferred because their values range between 0 and 1, although they can be negative if model errors are extremely large. In turn, RMSE values strongly depend on the scale of the independent variable. Therefore, it is not straightforward to set a limit on the RMSE value to accept or reject the model. Since *Q*_F*n*_^2^ values are always in the scale between 0 and 1, it is easy to set hard cut-offs for model selection, independently of the dataset or the magnitude of the target variable. However, this advantage is also its Achilles heel because it has led to an overuse of these metrics. Without any other parameter or additional analysis, *Q*_F*n*_^2^ could lead to wrong statements about the real predictivity of the model. The user should be aware of what *R*^2^ can and cannot say about the data. Several warnings regarding this parameter will be discussed below.

The equation to calculate *Q*_F*n*_^2^ includes a quotient where the numerator is the total sum of squares of the errors made by the model. If the total error is small, the quotient is also small and *Q*_F*n*_^2^ will be close to 1. Interestingly, discussion of the denominator in the formula and its effect on the *Q*_F*n*_^2^ value is often omitted.^72^ The denominator of the fraction is the deviation of the experimental data around the average activity or property either of the training or the test set. Thus, if the experimental activities are distributed over a large range of values, the quotient in the formula will be small too and the *Q*_F*n*_^2^ will be close to 1 as well. This means that if two models make exactly the same numerical error, their *Q*_F*n*_^2^ can be completely different if the molecules in each database have different activity ranges. In this regard, *Q*_F*n*_^2^ is not independent of the dataset and values from different datasets are not completely comparable. As an example, Table 3 shows internal and external RMSE and *R*^2^ values for several QSAR/QSPR models found in the literature. In these examples we use the symbol *R*^2^ as reported in the original papers. For instance, in the gas–ionic liquid partition coefficient reference (entry 2 in Table 3), the model with the lowest average error (RMSE = 0.068) also has the lowest *R*^2^ value (*R*^2^ = 0.791). The property range in this dataset (2.395–3.001) is smaller than in the other sets (0.209–2.248 and 0.836–2.569). If we had selected a model based on the *R*^2^ alone, we would have discarded the model with the lowest numerical error.

One of the most important implications of the dataset dependency of *Q*_F*n*_^2^ concerns the initial splitting of the database, particularly if the number of molecules is small. From the discussion above, the more different the distributions of the activities between the training and test sets are, the less comparable the *Q*_F*n*_^2^ values become.^73^ In other words, the same value will not have the same meaning if we are talking about the training or the test group. Thus, the context of the data is critical for decision making. In the example of the gas–ionic liquid partition coefficient models, we found that the discrepancy between the *R*^2^ and RMSE values could be attributed to the large differences on the property range between datasets. Following these ideas, setting hard cutoffs based on a single “universal” parameter is risky and could lead to misinterpretation or overconfidence in the model's performance. From our experience, the best practice when validating and reporting a QSAR/QSPR model is to report a set of statistics that support model predictivity accompanied by all the information required to understand the context of the data. Such information could be, for example, the activity distribution of both the training and test set using histograms or box plots and the regression plot of experimental *versus* predicted values. Although qualitative, the information gained from these visual representations of the data provides the appropriate context to interpret the numerical values of the statistics in a more meaningful way. Arguments stated above can also be extended to classification models and Table 3 also reports some parameters commonly used in its validation. Finally, Table 4 summarizes suggested methods that can help to contextualize the data.

**Table 4:** Suggested analysis for interpretation of validation parameters

Methods for description of data context	Interpretation
Histogram or box plot with the distribution of the independent variable of each dataset	The plot helps to compare the distribution of datasets and to compare their corresponding statistics
Regression plot (experimental *versus* predicted values for each dataset)	The plot provides a qualitative assessment of how experimental values are reproduced by the model. The importance of the regression plot is discussed and highlighted in ref. 74
Confusion matrix for classification models	Elements (*i*,*j*) of the confusion matrix show the number of molecules of class *i* that are predicted to belong to class *j*
Clustering or scaffold analysis	The method is useful to show how chemical populations are well represented in the training and validation sets
Consensus Diversity Plots (CDP)^12^ if several datasets are compared simultaneously	The CDP plot assists in the comparison of the diversity of datasets using different criterion. It approximates the global diversity of datasets
Principal component analysis (PCA) based on the variables in the selected model	PCA is useful to depict the distribution and density of molecules in the space of descriptors. Molecules in the validation sets should be inside the regions occupied by the training set

**Fig. 3:**
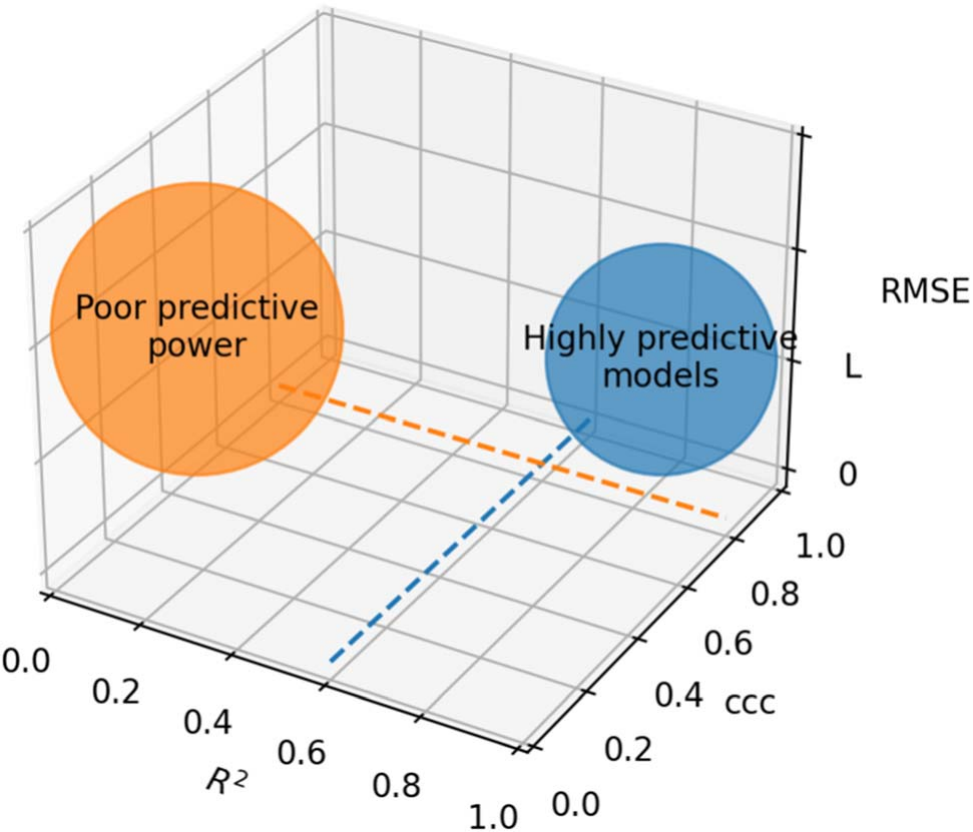
Schematic representation of optimal values for external validation parameters in predictive models. Since RMSE is dependent on the magnitude of activity values, a specific limit cannot be set. The case-dependent hypothetical limit for RMSE is represented as *L*.

Based on recommendations published before,^72,74,75^Table 5 summarizes exemplary validation statistics commonly used by the QSAR/QSPR community. Fig. 3 depicts a schematic map of expected values for highly predictive models. Clearly, since RMSE depends on the magnitude of the experimental activity, a general limit cannot be set. However, an RMSE value close to the experimental uncertainty can be regarded as an acceptable threshold for this metric. This hypothetical value is symbolized as *L* in Fig. 3.

**Table 5:** Internal and external validation statistics for selected and recently published QSAR/QSPR models

Endpoint	Training set(s) activity range(s)	Internal validation parameters	Test set(s) activity range(s)	External validation parameters	Reference
Intrinsic water solubility	−7.1 to −1.03	*R* ^2^ = 0.67	−6.79 to −1.18	*R* ^2^ = 0.42	76
		RMSE = 0.82		RMSE = 0.97	
				*R* ^2^ = 0.45	
				RMSE = 0.94	
				*R* ^2^ = 0.38	
				RMSE = 0.99	
	−8.8–1.7	*R* ^2^ = 0.62	−10.4 to −1.24	*R* ^2^ = 0.74	
		RMSE = 1.00		RMSE = 1.1	
		*R* ^2^ = 0.67		*R* ^2^ = 0.62	
		RMSE = 0.94		RMSE = 1.32	
				*R* ^2^ = 0.75	
				RMSE = 1.06	
Gas–ionic liquid partition coefficient	0.209–2.248	*R* ^2^ = 0.944	0.209–2.248	*R* ^2^ = 0.919	77
		RMSE = 0.092		RMSE = 0.101	
	0.836–2.569	*R* ^2^ = 0.915	0.836–2.569	*R* ^2^ = 0.891	
		RMSE = 0.102		RMSE = 0.110	
	2.395–3.001	*R* ^2^ = 0.791	2.395–3.001	*R* ^2^ = 0.717	
		RMSE = 0.068		RMSE = 0.072	
Impact sensitivity	0.70–2.51	*R* ^2^ = 0.89	0.70–2.22	*R* ^2^ = 0.84	78
		RMSE = 0.13		RMSE = 0.19	
	0.70–2.14	*R* ^2^ = 0.88	0.70–2.51	*R* ^2^ = 0.90	
		RMSE = 0.12		RMSE = 0.24	
Heat of decomposition	−2370 to −485	*R* ^2^ = 0.97	−2234 to −615	*R* ^2^ = 0.81	79
		RMSE = 99		RMSE = 301	
	−2370 to −485	*R* ^2^ = 0.95	−2234 to −615	*R* ^2^ = 0.68	
		RMSE = 117		RMSE = 345	
NOEC (no-observed-effect concentration) of polar and nonpolar narcosis	−9.93 to −2.180	*R* ^2^ = 0.76 s(residual) = 0.76	−4.61 to −0.055	*R* ^2^ = 0.727 s(residual) = 0.536	80
	−4.56 to −0.668	*R* ^2^ = 0.80 s(residual) = 0.49	−4.64 to −0.967	*R* ^2^ = 0.649 s(residual) = 0.402	
pIC_50_ for B-rapidly accelerated fibrosarcoma protein inhibitors	3.7–10.4	*R* ^2^ = 0.94	3.91–10.7	*R* ^2^ = 0.72	81
		MAE = 0.23		MAE = 0.52	
	3.7–10.4	*R* ^2^ = 0.84	3.91–10.7	*R* ^2^ = 0.53	
		MAE = 0.40		MAE = 0.67	
Water solubility	−13.17–1.58	*R* ^2^ = 0.9698	−12.78–2.40	*R* ^2^ = 0.7971	82
		RMSE = 0.4132		RMSE = 1.0355	
pIC_50_ of meprin β inhibitors	3.878–7.638	*R* ^2^ = 0.969	4.268–7.31	*R* ^2^ = 0.827	83
		RMSE = 0.189		RMSE = 0.411	
Corneal permeability coefficient	−6.17 to −4.1	*R* ^2^ = 0.9203	−4.33 to −6.85	*R* ^2^ = 0.8813	84
		MAE = 0.134		MAE = 0.214	
Aggregation number	4–113	*R* ^2^ = 0.9256	9–94	*R* ^2^ = 0.9526	85
		RMSE = 3.5268		RMSE = 0.0219	

## 3 Perspective

### 3.1 Vicious practices

Good practices in the development of QSAR models were reported in 2004 by Dearden.^86^ Such documentation serves as guidance for the advancement of the field. While some aspects have been steadily taken into consideration, others are inherently challenging to overcome. Notably, there are avoidable vicious practices that persist in the literature. As a community we should keep raising awareness on the benefits of following good practices and the risks of not doing so. The twenty-one errors when developing QSAR models, reported by Dearden *et al.* in 2004,^86^ were still valid, ten years later, as analyzed by Cherkasov *et al.* in 2014.^87^Table 6 shows the twenty-one errors classified as avoidable (17), partially/hardly avoidable (5). We should aim for reliable, transferable and, above all, useful models and not good but artifactual statistics or poorly performed models. When developing a model, one should make a conscious assessment of these errors, particularly the avoidable ones. Broader achievements and advancements in the field will come from the application of those models for predicting and testing new molecules, as well as on the continuous development of algorithms, analyses, and metrices to evaluate accuracy. Detailed description of each of these errors are described in the literature.^73,74^

**Table 6:** Twenty-one errors encounter when developing QSAR models^73^

Avoidable	Partially/hardly avoidable
Failure to take account of data heterogeneity	Use of incomprehensible descriptors
Use of inappropriate endpoint data	Error in descriptor values
Use of collinear descriptors	Lack of mechanistic interpretation
Poor transferability of QSAR/QSPR	Too narrow a range of endpoint values
Inadequate/undefined applicability domain	
Unacknowledged omission of data points/lack of activity cliffs analysis	
Use of inadequate data	
Replication of compounds in dataset	
Over-fitting of data	
Use of excessive numbers of descriptors	
Lack of/inadequate statistics	
Incorrect calculation	
Lack of descriptor auto-scaling	
Misuse/misinterpretation of statistics	
No consideration of distribution of residuals	
Inadequate training/test set selection	
Failure to validate a QSAR/QSPR correctly	

Most of the errors listed in Table 6 belong to the data curation step. Serious efforts have been made on the compilation and curation of databases. For example, errors in the generation of Tox21 are minimized by the careful design and collection of the data. It cannot be emphasized enough, the importance of the quality of the data when developing predictive models. Smaller datasets are more focused and are equally valuable, particularly if they are accompanied by external validation, those models are usually developed and published by academic researchers.

To advance in the quest for accurate predictive models, we must recognize and avoid the following:

Molecules considered for the external validation must be truly external. Preferably, the external validation set would contain molecules that would be synthesized or purchased and biologically evaluated. Else, a set of molecules with known biological activity are set aside and not used in model training. If those molecules are used in model training, they are just another internal validation set.

The selection of molecules used for building a predictive model should be carefully selected, particularly if local models are aimed. Whenever possible, local and global models should be built and compared, as they serve different purposes.^88^ If the molecules are too different, there is a risk that they might have different mechanisms of action. If the molecules are too similar, it is likely that the predictions made from that model will be highly accurate. However, the applicability domain will be rather limited. Thus, if the models are meant for design of new compounds or for regulatory purposes one should target high precision. If the predictive models are meant for screening purposes, less accurate models might be useful for considering diversity.

Data preparation is a central point to develop a useful model, however, normally its poorly performed. The identification, analysis, and elimination of activity cliffs and artifacts should be included. Since the data can be generated or gathered from different sources, the normality, dependance and colinearity of the data are relevant for the assessment of the suitability for modeling, and for the selection of the type of model to generate.^54^

The technology boom has allowed the construction of increasingly sophisticated and computationally demanding models. Such is the case of methods based on deep learning. However, most of the times the traditional methods offer better results than emerging predictive models. Thus, we discourage the selection of predictive models solely based on fashion.

Once the challenges are recognized, the researcher can consciously pursue better models and better science. Some aspects continue to be challenging, either because they are costly or too difficult to overcome. For example, as chemists we know the importance of stereochemistry (particularly in biological effects) but getting all possible stereoisomers and measuring its biological activity is, in most cases, too difficult. In several cases, the slight improvement in the model performance by taking into the consideration of the 3D-conformations justifies the use of simplest models based on 2D structure representations.

Since the lack stereochemical information will continue in the near future, one should keep in mind that essential information is missing and those molecules should not be included in the datasets for the development of predictive models.^87^ Building models ignoring stereochemistry is risky, more so for local than for global models.

### 3.2 Importance of documentation and open data, open models for assessment

Molecular design is becoming increasingly data-driven and can significantly improve its efficiency and effectiveness by implementing the FAIR (findable, accessible, interoperable, reusable) guiding principles.^89^ However, malpractices are slowing down the true impact that predictive models could have. For example, reporting a new methodology or model without providing the complete training data, or the exact methodology (code) for its reproduction. Fortunately, now data science initiatives are emerging to improve the reproducibility of reported predictive models and establish a benchmark for future models (*e.g.*, QSAR Databank).^90^ However, we must remember that it is important to carefully verify, clean, and analyze the initial datasets to analyze their applicability domain.

### 3.3 Leading examples

As a practical guideline to perform and report predictive models following good practices, we summarize representative examples in Table 7. Each case is different and require attention to specific steps on the process, however, good models can be performed and ultimately can be of use.

**Table 7:** Example of statistical parameters reported for validation

Endpoint	Statistical parameters	Reference
Hemato-toxicity	Sensitivity (SE)	91
	Specificity (SP)	
	Accuracy (ACC)	
	Balanced accuracy (BAC)	
	Matthew's correlation coefficient (MCC)	
	Area under the ROC curve (AUC)	
Toxicity	Accuracy (ACC)	92
	Sensitivity or true positive rate (TPR)	
	Fall-out or false positive rate (FPR)	
	Matthews correlation coefficient (MCC)	
	Receiver operating characteristic (ROC) analysis	
	Mean squared error (MSE)	
	Pearson correlation coefficient (PCC)	
Cruzain inhibitors	Leave-one-outcross-validation	93
	Coefficient of determination	
	Root-mean-squared error (RMSE)	
	Concordance correlation coefficient (CCC)	

A collection of published QSAR models is QSAR DataBank (QsarDB), freely available at https://qsardb.org/.^90^ QsarDB provides details on the datasets used, the methods employed, and the performance obtained for models for various application and research fields, including Chemical Sciences, Medical and Health Sciences, Biological Sciences, Environmental Sciences, Materials Sciences, Information and Computing Sciences, Mathematical Sciences, *etc.* This collection comes handy for the comparison of models.

### 3.4 Recent combined models

Non-quantitative models for the prediction of biological activity have been developed throughout the years, being the most notable one read-across, and its variants (CBRA^94^ and MuDRA^95^). Recently, the combined analysis of the read-across structure–activity relationships has been proposed^96^ along with the quantitative version (q-RASAR).^97^ Importantly, on that study, *R*^2^, *Q*^2^, *Q*_F1_^2^, *Q*_F2_^2^ and MAE were used for the assessment of the validation, predictive power, and error of the model developed. Thus, as the field moves forward on the development of predictive models, the metrics to assess accuracy continue to be an important element.

## 4 Conclusions

The availability of large amounts of data, and the development of algorithms makes possible the generation of predictive models. The easiness in the generation of certain types of data should be accompanied by careful design of datasets. Predictive models will continue to be useful, particularly when dealing with costly, time-consuming, or experimentally risky endpoints. Prediction of activities/properties ranges from relatively simple assays to more complex biological models such as animal systems and, ultimately, human beings. Areas of improvement are recognized and the associated errors or miss practices are and should be avoided.

Machine and deep learning are being instrumental to construct predictive and validated models of increased complex systems. Since the hype and extensive use of the artificial intelligence continues, a closer look at how this methodologies are influencing the accuracy of the predictive models is warranted. The availability of free and open resources, as well as the policies of peer-reviewed publications requiring the full publication of code and data, are largely facilitating or contributing to the contributions and applications of machine and deep learning to make predictive models. Less fortunate has been the influence of the hype of fashion so that there is an incremental number of publications on the predictive subject but with a lack of the rigor and required validation steps. In some instances, hype or fashion or lack of proper training and use of computational resources (or the easiness to access predictors), have contributed to carry out vicious practices that are still common.

As modelers we should care for data quality, reliability, and best practices. The ease of applying black-box methods to non-curated datasets without any reasoning is a recipe for failure. Moreover, it could propagate as potentially irreversible “tandem reaction” that ultimately will make credibility hard and fixing impossible.

Authors anticipate that over the next five to ten years there will be an explosion of raw data and predictive models and the models will be developed on large datasets. It remains to follow up on the quality of the data and the models themselves (validation with external data, or validated through their use). We also foresee that the number, variety, and quality of descriptors will continue to grow. It will remain to see its physical interpretability. Finally, we also anticipate a steady interest in the community to use the models that, most likely, will be translated in the job market of data science. Along these lines will be fundamental to enhance formal educational programs at universities for the proper training of developers and practitioners. Ideally, younger generations should be trained with a multidisciplinary approach such that they are aware not only in the rigor of the model development but also in the practical application and use of the models.

## Author contributions

K. G. P., J. G. R. J. and E. L. L. collected data and generated the figures, K. M. M. and J. L. M. F. designed the manuscript, and all the authors contributed to its writing.

## Conflicts of interest

There are no conflicts to declare.

## Supplementary Material
